# Use of two new formulations as bovine embryo manipulation solution

**DOI:** 10.21451/1984-3143-AR2018-0044

**Published:** 2019-10-24

**Authors:** Flavio Guisseli Lopes, Eduardo Paulino da Costa, Vanessa Lopes Dias Queiroz-Castro, Emilio Cesar Martins Pereira, José Domingos Guimarães, Saullo Vinicius Pereira Alves, Carlos Antonio Carvalho Fernandes, Luiz Sérgio Almeida Camargo, Laercio dos Anjos Benjamim

**Affiliations:** 1 Veterinary Department, Federal University of Vicosa, Peter Henry Rolfs Avenue, Viçosa, Minas Gerais, Brazil.; 2 José do Rosário Vellano University (UNIFENAS), Alfenas, Minas Gerais, Brazil.; 3 Embrapa Gado de Leite, Juiz de Fora, Minas Gerais, Brazil.

**Keywords:** cell apoptosis, Hsp 70.3, mice

## Abstract

This study aimed to evaluate the effect of two Embryo Manipulation Solutions (EMS and EMS supplemented) in maintenance of the viability of embryos, initially using structures derived from mice (first phase). Next, the efficiency of these solutions in routines of bovine embryo transfer was evaluated (second stage). Mice embryos were used in the stages of early blastocyst, and compact morula grades I and II. These embryos were initially randomly distributed and maintained for four hours in three solutions: Modified phosphate buffered saline (PBS; Control); EMS (treatment 1), and EMS supplemented (treatment 2). Subsequently, they were cultured in TCM 199 medium and evaluated in terms of total number of cells, morphometric characteristics, ultra structural aspects, detection of cell apoptosis, and quantification of *Hsp*70.3 gene expression. In the second phase, these same solutions were tested in the transfer of quality I and II bovine embryos (excellent and good). These embryos were transferred fresh to 58 recipients. The results showed that the total number of cells in embryos expanded blastocyst (ExB), the number of apoptotic cells, the cell, nuclear, nucleolar diameter and the nucleus/nucleolus ratio was similar among the treatments. The pregnancy rate shown on second phase was also similar. However, the EMS supplemented expressed more *Hsp70.3* than EMS. The expression of *Hsp70.3* was also greater for embryos in EMS than that of EMS supplemented. The McII embryos, EMS and EMS supplemented samples also expressed more *Hsp70.3* compared to control embryos. In conclusion, the tested solutions can be used in routine embryo transfer techniques, replacing modified PBS solution as an effective media in maintaining embryo viability.

## Introduction

The improvement of the techniques involved in the process of *in vitro* production (IVP) of embryos is of fundamental importance to the study and understanding of biological mechanisms underlying embryonic development. The choice of media and energy substrates has an important impact on the development and viability of embryos (Vanroose *et al*., 2001).

According to Donnay and Leese (1999) energetic substrates, such as piruvate, glucose, lactate and amino acids play an important role in embryo development, explaining the use of different embryo manipulation and culture media.

During embryonic development, specifically in the preimplantation phase, genes are expressed in the blastocyst stage that are responsible for the processes that occur during cell differentiation and the implantation phase, an event that has a high percentage of embryonic mortality (Ponsuksili *et al*., 2002). Thus, any modification in culture conditions could directly affect embryo quality (Rizos *et al*., 2003). Even brief exposure to the manipulation medium, an average period of four hours, is sufficient for the occurrence of molecular and morphological changes (Gordon, 1994; Bavister, 1995). There are evidences showing that culture conditions have an impact on pre and post-implantation development and possibly the future health of the offspring (El Hajj and Haaf, 2013; Mantikou *et al.*, 2013).

Increase in the number of apoptotic cells, alteration of gene expression, and ultra-structural modifications are examples of these changes (Rizos *et al*., 2003; Lonergan *et al*., 2003). Apoptosis, despite being regarded as a normal process in embryos during the preimplantation stage (Byrne *et al*., 1999), when occurs excessively, can lead to impairment of embryonic development (Levy *et al*., 2001). The proteins belonging to the family of the Hsp of 70 kilodaltons (kDa) is expressed as a reflection of cell stress (Pedersen *et al*., 2005). This protein is one of the first to be produced during embryonic development and is of great importance for cell function (Zhang *et al*., 2011). According to Lane and Gardner (1997), normal fetal development is dependent on the total number of cells in the embryo. Mori *et al*. (2002) and Hoelker *et al*. (2006) reported a positive correlation between the total number of cells and diameter of bovine embryos produced *in vitro*. Thus, the total number of cells is frequently used as a parameter to evaluate the quality of embryos (Viuff *et al*., 2001; Pereira *et al*., 2005; Costa *et al*., 2010).

The manipulation solution routinely employed in conventional embryo transfer (ET) in cattle is modified phosphate buffered saline (PBS) (Whittingham, 1971).

According to the success obtained by Whittingham (1971), few studies have been carried out in order to find more appropriate and stable solutions for the manipulation of embryos in different animal species. However, modified PBS is subject to possible changes in composition, such as pH variations, formation of precipitates of calcium chloride and magnesium chloride, especially when stored for a long time in ambient temperature and light (Gordon, 1994; IETS, 1998). Therefore, this media should be stored in a low light, in a refrigerated environment (minimum temperature of 4ºC), in order to prevent degradation of some components such as pyruvate (Taylor, 1984).

Considering these aspects, it would be desirable to use a media that is able to maintain the viability of the embryos, more stable, with minimum possibility of alteration during storage, even at room temperature. In view of this fact, the medium tested in the present study met these requirements, being therefore a more appropriate medium for use, since its stability.

The present study was designed to evaluate cleavage rate, diameter, morphological aspects, cell apoptosis, and gene expression of *Hsp70.3* in mice embryos, after maintenance in different manipulation solutions and subsequent *in vitro* culture. At a later stage, bovine embryos were manipulated in these solutions and transferred to the bovine recipients, and we evaluated pregnancy rate to determine if it was feasible to substitute the media conventionally used for simpler and more stable solutions that could be stored at room temperature.

## Material and Methods

The experiment was conducted in two phases to evaluate the effects of embryo manipulation solutions. In Phase I, mice were used, which are commonly used as an experimental model in studies of *in vitro* embryo production. The solutions that maintained embryo viability in this phase were tested later in bovine embryos, which constituted Phase II.

Phase I was conducted at the Animal Reproduction Laboratory of the Federal University of Vicosa (UFV), using mice embryos of *Mus musculus* species, of the Swiss albino strain. Phase II involved bovine embryos produced *in vivo* that were maintained in the tested solutions and subsequently inoculated in bovine recipients.

All experimental procedures were performed in accordance with the ethical principles adopted by the National Research Council, upon authorization from the Animal Ethics Committee of UFV, processed 10/2006.

### Phase I

Two manipulation solutions used for collection and manipulation of embryos were tested: an embryo manipulation solution (EMS): sodium chloride (0.1027 mol L^-1^), potassium chloride (0.0040 mol L^-1^), calcium chloride (0.0018 mol L^-1^); sodium lactate (0.0277 mol L^-1^); and EMS supplemented (the same formulation of EMS plus 0.0003 mol L^-1^ sodium pyruvate). The modified PBS served as the control (Whittingham, 1971).

The EMS and EMS supplemented solutions were produced and adjusted with glucose to 290 mOsm with the aid of an osmometer (Osmette A® Automatic Osmometer) and the pH was adjusted to 7.2-7.4. All reagents used for the production of manipulation and culture solutions were previously tested for cell culture and were from Sigma Chemical Co.® (St. Louis, MO). The antibiotics used for EMS and EMS supplemented solutions were the same as used in the modified PBS (Whittingham, 1971), in which 50mg Streptomycin sulfate and 100UI/mL of G Sodic-Penicillin were added.

Following the conventional superovulation protocol (Rafferty, 1970), mice embryo donors were induced to superovulation and mated. After 60-80 h following mating, the donor females were euthanized for collection of embryos, which was accomplished by washing the uterine horns. The media tested were used for the washing.

Only embryos in the stages of early blastocyst (EB), and compact morula grades I (McI) and II (McII) were used, and were randomly distributed into three treatments: T1 - modified PBS (Control); T2 (EMS); and T3 (EMS supplemented). The embryos from each treatment were maintained in four-well plates (Nunc® A/S), for four h, in the manipulating solutions at 37°C plus 0.4% bovine serum albumin, fraction V.

After the end of the manipulation period, the embryos were classified according to their stage of development and embryo quality was assessed (IETS, 1998) using a stereoscopic microscope (Olympus Optical®, SZ-40 model/SZ-ST) with a 10X eyepiece and 4X objective, which was used throughout the experiment. After being classified, embryos were transferred to media and cultured in four-well plates (Nunc® A/S), for 10h in a modified TCM-199 culture medium (Costa *et al*., 1997) in an incubator oven (Jouan®, IG 150 Model) at 37°C in an atmosphere of 5% carbon dioxide, 95% atmospheric air, and at 95% humidity.

Following the culture period, the embryos were transferred and washed in a hollowed plate in modified Talp-Hepes medium at 37°C (Costa *et al*., 1997), and were classified again. After the culture, 70 embryos in the initial stage of expanded blastocyst (ExB) were randomly separated and submitted to morphometric evaluation in accordance with the methodology of Fukui and Ono (1989), with the aid of the stereoscopic microscope.

This evaluation was carried out in three periods with the aid of the same microscope, which had a micrometric reticulum (1 mm per side with 100 subdivisions) in the 10X eyepiece and 4X objective. The diameter of embryos at the time of collection, at the end of the manipulation period, and at the end of the culture period was recorded. After taking measurements, the means and standard deviations were calculated using the appropriate correction factor for the determined increase (Hoffmann, 1987).

The procedures of Costa (1994) were adopted for evaluation using transmission electron microscopy (TEM). For each treatment, five embryos were used, which were in ExB stage following the culture period. After the inclusion of the embryos (Costa, 1994), semi-thin sections (0.5 μm) were obtained using a glass knife on an ultramicrotome. The best sections were selected under optical microscopy to perform the ultrathin sections (60-70 nm) using a diamond knife. These ultrathin sections were stained with uranyl acetate and lead citrate and evaluated using TEM (Zeiss^®^, EM 109 model).

For the detection of cell apoptosis and to determine the total cell number, the TUNEL (Terminal Transferase Mediated Uracil Nick End Labeling) technique and the APO-BrdU^TM^ Assay Kit (Invitrogen^®^, CA, USA) were used. For each treatment, five embryos were used, which were in the ExB stage after culture. The embryos from each treatment were fixed on slides and processed according to the manufacturer’s instructions (Promega®, WI, USA). The slides were analyzed on a fluorescence microscope and observed through a filter (520 ± 20 nm) with a 40X objective. Cells that were green were considered TUNEL-positive cells (i.e., apoptotic cells). In this step, the total number of cells present in the embryos was also counted.

Quantitative variables (TEM and cell apoptosis) were submitted to tests for normality (Lilliefors) and homoscedasticity (Cochran), and subsequently to an analysis of variance (ANOVA). When significance was detected, Duncan’s comparisons of means test was carried out. When the assumptions of normality and homoscedasticity were not attained after the appropriate transformations, data were submitted to the nonparametric Kruskal–Wallis test (UFV, 1999).

For the quantification of gene expression, the RT-PCR technique was used in real time, evaluating two genes, one endogenous (BActin) and one related to heat stress (*Hsp70.3*), as summarized in Tab. 1. For each manipulation solution, tested three samples of embryos (10 in each sample), in duplicate, classified at the time of collection in EB, McI, and McII stages, totaling 30 embryos per treatment. After the end of the culture period, the embryos that were in the ExB stage were also evaluated.

**Table 1:** Sequence of primers and annealing temperatures specific to each gene evaluated.

Genes		*Primers* (5’-3’ sequence)	AT (ºC)
BActin	Forward	5’ TCTTGGGTATGGAATCCTGTGGCA 3’	60,1
	Reverse	5’ AGATGTGGATCAGCAAGCAGGAGT 3’	59,5
*Hsp70.3*	Forward	5’ CGCTCGAATCCTATGCCTTCAACA 3’	58,9
	Reverse	5’ GCACTTGTCCAGCACCTTCTTCTT 3’	59,4

AT: Annealing temperature.

The RT-PCR reactions in real time were carried out in an ABI Prism 7300 Sequence Detection Systems thermocycler (Applied Biosystems, CA, USA), using SYBR Green^®^ PCR Master Mix Kit (Applied Biosystems, CA, USA), according to the manufacturer’s recommendations. The reactions contained 12.5 µL PCR MIX, 5 µL cDNA, primer, and water, totaling 25 µL. The concentrations of primer and cDNA were previously optimized for each gene.

Data obtained were analyzed by REST^®^ program (Pfaffl *et al*., 2002), available at http://www.wzw.tum.de/gene-quantification, which uses the Pair Wise Fixed Reallocation Randomization Test^®^ model to compare expression between the samples from the treatments.

### Phase II

The solutions that maintained mice embryo viability were tested later in bovine embryos. Fifty-eight crossbred heifers with body weight >350 kg were used as recipients of embryos. Throughout the experimental period, the animals were maintained in paddocks with *Brachiaria brizantha* grass, mineral supplementation, and water *ad libitum*. All heifers that were between days 7 and 17 of the estrous cycle, or had the corpus luteum previously detected by ultrasound or transrectal palpation, received 0.53 mg sodium cloprostenol (Ciosin^®^; Schering-Plough Brazil Ltd, Brazil) through intramuscular injection (IM). This medication was administered to the recipients 24h before the application of the donors during the process of superovulation. The recipients that expressed estrus 24h before and through 24h after the donors were considered synchronized and suitable for embryo transfer.

Four Holstein cows with body weight >500 kg were used as embryo donors. Animals that exhibited estrous cycles with regular intervals (21 ± 3 days) and were clinically normal on gynecological examination were selected. The animals received the same diet throughout the experimental period. The animals were maintained in paddocks of *Brachiaria brizantha* grass, in the presence of a ruffian, with mineral supplementation and water *ad libitum*.

Treatment of the donors initiated on predetermined days, with the application of a progesterone intravaginal device (CIDR®; Pfizer Animal Health, Brazil). On the following day, 3 mg estradiol benzoate (RIC-BE®; Syntex S.A.; Argentina) was administrated via IM injection.

Five days following the application of the progesterone device, the superovulation protocol was initiated with the administration of decreasing doses of FSH-p hormone (Folltropin-V®; Vetrepharm Inc.; Canada) via the IM route at intervals of 12h for a total of eight applications (total concentration from 140 to 200 IU). With the seventh application 0.53 mg sodium cloprostenol (Ciosin®; Schering-Plough Brazil Ltd, Brazil) was applied to induce luteolysis. At the eighth application, the progesterone intravaginal device was removed. After the detection of estrus, two inseminations were performed, the first between 10 and 12h, and the second between 20 and 24h after the onset of estrus.

Embryos were collected by non-surgical method between the sixth and eighth day following the onset of estrus. The manipulation solutions to be evaluated were preheated to 37°C and used to wash the uterine horns.

In the farm laboratory, we searched for embryos with the aid of a stereoscopic microscope (Olympus Optical®, SZH-ILLB model) with 15X magnification. Once found embryos were classified according to stage of development and embryo quality (IETS, 1998) and were transferred to dish and maintained for a maximum period of four hours. The manipulation solutions were EMS supplemented, EMS, and modified PBS, and were previously equilibrated (37°C) with the addition of 0.4% bovine serum albumin, fraction V.

The ET was carried out fresh (i.e., the embryos were not frozen). Only quality I and II embryos (excellent and good) were collected and transferred. The recipients were randomly distributed into three treatments: T1 – modified PBS (Control), T2 (EMS), and T3 (EMS supplemented). Embryo inovulation was performed by the transcervical method.

Pregnancy diagnosis was performed on the 28th day after detection of estrus in the recipients through ultrasonography (Pie Medical®, Scanner 100 Falco model), connected to a transducer for 6/8 MHz bifrequencial endorectal evaluation. For statistical analysis of pregnancy rate, the data were compared in contingency tables and analyzed by chi-square test at a 5% significance level (Sampaio, 2002).

## Results

### Phase I

A total of 1.542 mice embryos were recovered and sorted between the experiments. The total number of cells in embryos in the ExB stage was similar among the treatments (P > 0.05), as shown in Table 2. The mean diameter of the embryos in EB stage showed a higher growth rate (P < 0.05) when maintained for a period of four hours in the EMS and EMS supplemented solutions, compared to the control group (modified PBS) (Tab. 3). However, the embryos in McI and McII stages showed no increase in diameter (P > 0.05; Tab. 3).

**Table 2:** Means (± standard deviations) of the total number of cells in mice embryos in the expanded blastocyst stage after the maintenance in manipulation solution and subsequent *in vitro* culture, in the different treatments.

Treatments	Total of embryos (N)	X ± SD
Control	35	54.1 ± 10.7
EMS	35	55.5 ± 11.4
EMS supplemented	35	53.2 ± 9.6

Control: modified PBS (Whittingham, 1971). X = mean; SD = standard deviation. The differences were not significant (P > 0.05) among the treatments according to the Duncan test.

**Table 3:** Diameter (micra) of mice embryos in early blastocyst, compact morula grades I and II stages after the collection, maintenance in manipulation solution, and subsequent *in vitro* culture, in the different treatments.

Treatments	Collection (0 h) X ± SD	Maintenance (4 h) X ± SD	Culture (10 h) X ± SD
(EB)			
Control	73.7 ± 7.8^a,A^	74.4 ± 7.0^a,A^	96.7 ± 10.5^a,B^
EMS	74.4 ± 8.3^a,A^	76.5 ± 7.7^a,b,A^	97.4 ± 10.8^a,B^
EMS supplemented	74.7 ± 7.2^a,A^	78.9 ±7.3^b,A^	97.7 ± 9.3^a,B^
(McI)			
Control	71.7 ± 4.6^a,A^	71.3 ± 5.0^a,A^	90.2 ± 8.9^a,B^
EMS	71.7 ± 5.4^a,A^	73.0 ± 6.7ª^,A^	91.2 ± 8.3^a,B^
EMS supplemented	71.3 ± 5.0^ª,A^	73.7 ± 5.9^a,A^	92.6 ± 6.9^a,B^
(McII)			
Controle	71.0 ± 6.1^a,A^	71.7 ± 5.4^a,A^	84.0 ± 10.5^a,B^
EMS	71.0 ± 5.3^a,A^	73.0 ± 6.1^a,A^	86.4 ± 10.0^a,B^
EMS supplemented	71.7 ± 5.4^a,A^	72.0 ± 5.0^a,A^	86.1 ± 9.4^a,B^

Control: modified PBS (Whittingham, 1971). X = mean; SD = standard deviation; EB: early blastocyst; McI: grade I compact morula; McII: grade II compact morula. Means with different lowercase letters in the same column indicate difference (P < 0.05) among treatments for the EB, McI (Tuckey test) and McII (Kruskal-Wallis test) stages. Means with different uppercase letters in the same line indicate difference (P < 0.05) among treatments for the EB (Tuckey test), McI and McII (Kruskal-Wallis test) stages.

No difference in the cell, nuclear, nucleolar diameters and or the nucleus/nucleolus ratio was observed even after the maintenance for 4 h in the tested manipulation solutions (P > 0.05), as compared to the control group (Tab. 4). No difference was observed among the treatments (P > 0.05) in the number of apoptotic cells. Means and standard deviations for the different treatments were 38.0 ± 10.7 (control), 37.1 ± 11.4 (EMS), and 40.0 ± 9.6 (EMS supplemented).

**Table 4:** Means (± standard deviations in micras) of the cell diameter, nuclear diameter, nucleolar diameter, and nucleus/nucleolus ratio in embryos in the early blastocyst stage after manipulation and subsequent *in vitro* culture in the different treatments.

	CD	ND	NLD	NNR
Treatment	X ± SD	X ± SD	X ± SD	X ± SD
Control	15.0 ± 7.1	6.6 ± 1.7	2.4 ± 0.6	2.8 ± 0.4
EMS	25.6 ± 10.5	9.8 ± 7.1	2.5 ± 0.3	3.8 ± 2.5
EMS supplemented	17.3 ± 7.6	6.5 ± 1.7	1.7 ± 0.6	3.9 ± 0.4

Control: modified PBS (Whittingham, 1971). X = mean; SD = standard deviation. CD: cell diameter, ND: nuclear diameter, NLD: nucleolar diameter; NNR: nucleus/nucleolus ratio; EB: early blastocyst; McI: compact morula grade I; McII: compact morula grade II. The differences were not significant (P > 0.05) among the treatments by the Duncan test.

With the aid of REST® software, it was possible to perform comparisons among the treatments for gene expression of *Hsp70.3* (Tab. 5). Considering the EB embryos, the EMS and EMS supplemented solutions expressed more *Hsp70.3* than did control embryos (P < 0.001). It was also found that EMS supplemented expressed 9.071 times more *Hsp70.3* than EMS (P < 0.001). Similarly, for McI embryos, EMS and EMS supplemented samples expressed more than control samples (P < 0.001). The expression of *Hsp70.3* was 3.007 times greater for embryos in EMS (P < 0.001) than that of EMS supplemented. The McII embryos, EMS and EMS supplemented samples also expressed more *Hsp70.3* compared to control embryos (P < 0.001). A higher expression was observed for EMS supplemented than EMS (P < 0.001).

**Table 5:** Relative expression of *Hsp 70.3* gene among the samples of the treatments.

		Relative expression		
Gene	Samples	Cnt *vs* EMS	Cnt *vs* EMS suppl.	EMS *vs* EMS suppl.
*Hsp70.3*	EB	1,421^a^	12,889^b^	9,071^c^
	McI	4,129^a^	1,373^b^	3,007^c^
	McII	20,959^a^	9,217^b^	2,274^c^

Means with different lowercase letters in the same line indicate difference among treatments (P < 0.05) by REST^®^ software. Cnt = Control: modified PBS of Whittingham (1971).

### Phase II

No difference among treatments was found for pregnancy rate (P > 0.05; Table 6).

**Table 6:** Pregnancy rates in bovine recipients inoculated with fresh embryos, in the different treatments.

Treatments	N	Pregnants (N)	Pregnancy (%)
Control^1^	19	9	47.4^a^
EMS	19	9	47.4^a^
EMS supplemented	20	11	55.0^a^

1 Control: modified PBS (Whittingham, 1971). N = number of animals. There was no difference among treatments (P > 0.05), by the Chi-square test.

## Discussion

The number of cells described in this study is similar that of previous studies. Alexandre (1978) and Evsikov (1996) reported cell means for cleavage rates of mouse embryos produced *in vitro* of 46.6 ± 2.7 and 60.5 ± 1.4, respectively. However, *in vivo*, Bowman and Mclaren (1970) and Smith and Mclaren (1977) observed cell means of 56.9 ± 7.81 and 44.8 ± 3.2, respectively. These data emphasize the influence that manipulation and culture media have on the development and quality of embryos.

Thus, it can be affirmed that the solutions tested (EMS and EMS supplemented) maintained the cleavage rate and consequently, the quality of embryos after four hours of manipulation. According to Lane and Gardner (1997), normal fetal development is dependent on the number of total cells of the embryo. Zhu *et al*. (2014) emphasize in their research the importance of this parameter since they found that grade III or IV embryos with 7-8 cells were better than grade I or II embryos with only 4 or 5 cells.

In the present experiment, the mean diameter presented by EB embryos, McI, and McII at the time of collection, at the end of the period of manipulation, and at the end of *in vitro* culture were within the normal range, in agreement with Bowman and Mclaren (1970) and Chung (1973). According to Lindner and Wright (1983), embryo diameter is the same during the morula and blastocyst stages, and increase occurs only in the expanded blastocyst stage, at which time there is a considerable increase in the diameter (1.2-1.5 times), and the zona pellucida is decreased by 1/3 of its original thickness.

According to Rieger (1992) and Martin and Leese (1995), the pyruvate captured by the embryo presents different percentages of metabolization, in accordance with the phase of development. According to these authors, 44% is metabolized in the two-cell stage and 17% in the morula stage. After which, glucose becomes the substrate predominantly used. This fact validates the results of the present study, in which the size of the morula stage in the EMS and EMS supplemented treatments did not differ from that of the control (modified PBS). Thus, it is probable that the pyruvate made available by the tested solutions was not been fully used by the embryos in McI and McII. In addition, both solutions presented similar results in relation to the control, revealing them sufficient to maintain embryonic development at ideal rates.

However, the rate between pyruvate: lactate is essential for the balance of the oxidation/reduction potential (Morales *et al*., 1999) Butcher *et al*. (1998) also suggest that the pyruvate could be converted into alanine and act to remove ammonia from the embryo, playing a role in embryo protection. In addition, it acts degrading the exogenous hydrogen peroxide (H_2_O_2_) and preventing the embryo from oxidative stress (Morales *et al*., 1999), which may justify the supplementation of pyruvate in the manipulation media.

The results of this study suggest that the tested solutions were able to promote sufficient metabolic stimuli to maintain the morphological characteristics of the embryos under normal conditions because the results were similar to the solution used in the control group. According to Knijn *et al*. (2003), the incidence of apoptosis was higher in embryos produced *in vitro* than in those produced *in vivo*, because embryos are subjected to adverse conditions, such as exposure to high culture temperatures for 7 days. Therefore, the increased incidence of cell death is an important indicator of embryo quality (Vanda Ele *et al*., 2007).

The manipulation media EMS and EMS supplemented were not able to reduce the number of apoptotic cells compared to the control group. Few studies in the literature separately report the incidence of apoptosis in early blastocyst, blastocyst, and expanded blastocyst stages; however, it is known that the largest quantity of apoptotic cells is found in the expanded blastocyst stage (Fabian *et al*., 2005; Fabian *et al*., 2007).

The results demonstrated a difference in the *Hsp 70.3p* expressed in EB, McI, and McII embryos after maintenance and subsequent *in vitro* culture in the different treatments. According to Lonergan *et al*. (2003), embryonic gene expression can be modified in response to environmental changes, which is probably an attempt of the embryo to stabilize its cellular function. Thus, the observed differences can be related to the use of the manipulation solutions. The culture medium is one of the factors that can modify the transcription activity of the Hsp genes (Sagirkaya *et al*., 2006; Lazzari *et al*., 2002).

Exposure to stressful situations results in morphological changes in the embryo. The redistribution of organelles in the cortical region, cytoplasmic aggregation, and vacuolization and rupture of the mitochondrial membrane and/or matrix can result in apoptosis and cell death (Li *et al*., 2000). Although an increased expression of *Hsp70.3* in embryos maintained in EMS and EMS supplemented occurred, there was no impairment of embryonic development. Machado *et al*. (2013) observed that among the evaluated genes related to stress only *Hsp 70* was different between *in vivo* and *in vitro* system and, unexpectedly, the *in vivo* system presented the highest quantity of Hsp. The authors pointed out that the *Hsp 70* mRNA of the *in vitro* embryos in adverse conditions might be depleted more quickly in order to produce the protein in face of the stressful conditions. The EMS and EMS supplemented solutions are composed of energy substrates that have a positive effect on embryo development, such as glucose, lactate, and pyruvate (Dorland *et al*., 1992; Thompson, 2000; Riley and Moley, 2006).

Working with mice embryos, Beckmann *et al*. (1992) and Dix *et al*. (1998) found that minimum levels of *Hsp70.1* and *Hsp70.3* are necessary for the normal development of preimplantation embryogenesis. This may have occurred in the present study, because despite the increased expression of *Hsp70.3* in the treatments, the characteristics of morphology and apoptosis of the embryos were not affected. This result is contrary to the findings of Fear and Hansen (2011), who indicated that in bovine embryos, the increase of *Hsp* demonstrated association with apoptosis and consequently, embryo death.

The pregnancy rates obtained in this experiment were similar to those observed by several researchers using the modified PBS for the manipulation of bovine embryos. Macmillan (1998) searched the published literature regarding bovine fresh ET and found an average of 43% pregnancy. Similar results were reported by Gioso *et al*. (2005) and Haas *et al*. (2007), who found pregnancy rates of 45.5% and 50%, respectively.

The positive results are supported by the composition of the EMS and EMS supplemented solutions, which contain energy substrates that provide conditions for the normal development of the embryos. The EMS solution has glucose and lactate, whereas the EMS supplemented contains three energy substrates: glucose, lactate, and pyruvate.

The use of pyruvate was proposed because it is used by embryos during their development (Thompson, 2000). After blastulation, there is an increase the need for pyruvate, the lack of which could interfere with the viability of the blastocyst (Dorland *et al*., 1992).

Lactate is another component that allows embryo development from the single cell stage to the blastocyst (Bavister, 1995). Its interaction with pyruvate was described as essential for the balance of the oxidation/reduction potentials (Morales *et al*., 1999). The presence of glucose in the manipulation solution is justified by the necessity of its incorporation in the final stages of embryonic development. The greater consumption of glucose at this stage occurs in most species embryos IVP systems, a glucose uptake facilitates the production of better quality embryos reflecting in successful transfers. (Thompson, 2000; Gardner and Wale, 2013; Absalón-Medina *et al*., 2014).

The interaction of these substrates, contained in the proposed manipulation solutions, may have been responsible for the development of embryos, and consequently, for the satisfactory pregnancy rates found in this study.

## Conclusion

The manipulation solutions (EMS and EMS supplemented) did not interfere in embryo cleavage rate, and can be used as a substitute for the modified PBS solution. It was concluded that the EMS supplemented solution favors the greater development of EB after the period of manipulation and subsequent *in vitro* culture. Although the solutions result in expression of a greater amount of *Hsp70.3,* they maintain the ultrastructural characteristics and the normal proportion of apoptotic cells of the embryos.
